# Alternative expression of TCRζ related genes in patients with chronic myeloid leukemia

**DOI:** 10.1186/1756-8722-5-74

**Published:** 2012-12-10

**Authors:** Xianfeng Zha, Xiaojuan Yan, Qi Shen, Yuping Zhang, Xiuli Wu, Shaohua Chen, Bo Li, Lijian Yang, Suxia Geng, Jianyu Weng, Xin Du, Yangqiu Li

**Affiliations:** 1 Institute of Hematology, Medical College, Jinan University, Guangzhou, 510632, China; 2 Department of Hematology, Guangzhou First Municipal People’s Hospital Affiliated to Guangzhou Medical College, Guangzhou, 510180, People’s Republic of China; 3 Department of Hematology, Guangdong General Hospital (Guangdong Academy of Medical Sciences), Guangzhou, 510080, China; 4 Key Laboratory for Regenerative Medicine of Ministry of Education, Jinan University, Guangzhou, 510632, China

**Keywords:** ASF/SF-2gene, TCRζ3^′^-UTR, TCRζ gene, FcεRIγ gene, Chronic myeloid leukemia, Real-time PCR

## Abstract

A previous study has demonstrated a significant decrease in the TCRζ gene expression level in chronic myeloid leukemia (CML); thus, we further investigated the expression of TCRζ-regulating factors, the distribution of the TCRζ 3' untranslated region (3'-UTR) splice variants, and the expression level and correlation of the alternative splicing factor/splicing factor 2 (ASF/SF-2), FcεRIγ and ZAP-70 genes. TCRζ 3'-UTR splice variants were identified in peripheral blood mononuclear cells (PBMCs) from 14 healthy individuals, 40 patients with CML and 22 patients with CML in complete remission (CML-CR) by RT-PCR. The expression level of the TCRζ, FcεRIγ, ASF/SF-2 and ZAP-70 genes was analyzed by real-time quantitative PCR. While the expression of TCRζ gene in the CML group was significantly lower than that in the healthy individual and CML-CR groups, a significantly higher expression of the FceRIγ and ASF/SF-2 genes was found in the CML group. Two types of splicing forms were detected in all of the healthy individual CML-CR cases: wild type (WT) TCRζ 3'-UTR and alternatively splieced (AS) TCRζ 3'-UTR which have been alternatively splieced in the WT TCRζ 3'-UTR . However, 35% of the CML cases contained only the wild type TCRζ 3'-UTR isoform. Based on the TCRζ 3'-UTR isoform expression characteristic, we divided the patients with CML into two subgroups: the WT^+^AS^-^ CML group, containing patients that express only the wild type TCRζ 3'-UTR, and the WT^+^AS^+^ CML group, which contained patients that expressed two TCRζ 3'-UTR isoforms. A significantly different ASF/SF-2 and FcεRIγ gene expression pattern was found between the WT^+^AS^-^ and WT^+^AS^+^CML groups. We concluded that defective TCRζ expression may be characterized in the WT^+^AS^-^and WT^+^AS^+^CML subgroups by the different gene expression pattern. The overexpression of ASF/SF2, which alternatively splices the TCRζ 3’-UTR, is thought to participate in feedback regulation. The characteristics of TCRζ 3'-UTR alternative splicing may be a novel immunological marker for the evaluation of the CML immune status.

## Introduction

Chronic myeloid leukemia (CML) is a clonal hematopoietic stem cell disease that is characterized by the Philadelphia chromosome (Ph), which is generated by the reciprocal translocation t(9;22)(q34;q11) that results in the fusion of the c-abl oncogene 1 (ABL1) with the breakpoint cluster region (BCR) gene [1]. T cell immunodeficiency including thymic output function, abnormal T cell receptor (TCR) repertoire expression and, in part, abnormal TCR signal transduction, such as that involving the TCRζ chain is found in patients with CML [2-6], and T cell function becomes suppressed as the disease progresses in some patients.

The TCR/CD3 complex plays a central role in T cell activation. This complex comprises of two chains, αβ or γδ, these chains are noncovalently associated with CD3 subunits, which include four transmembrane proteins: CD3γ, CD3δ, CD3ε and CD3ζ (also referred to as TCRζ). These subunits are known to form three distinct dimers, CD3γε, CD3δε, and CD3ζζ, to mediate TCR signal transduction [7-10]. There are four tyrosine kinase families involved in TCR signal transduction including the Csk, Src, Tec, and ZAP-70 (CD3 zeta chain associated protein kinase 70 kDa) kinase families [11]. ZAP-70 is a cytosolic protein that is recruited to the T cell plasma membrane following TCR stimulation and binds to phosphorylated TCRζ immunoreceptor tyrosine-based activation motifs (ITAMs); it plays a critical role in activating downstream T cell signal transduction pathways following TCR engagement [11]. There is evidence that the Fce receptor type Iγ (FcεRIγ) chain, which is a member of the TCRζ chain protein family and a component of the high-affinity IgE receptor, can replace a functionally deficient TCRζ chain and facilitate TCR/CD3 complex-mediated signaling [12,13].

The absence of the TCRζ chain not only influences the TCR expression on the cell membrane and the number of single positive (i.e., CD4+ or CD8+) circulating T cells, it also impairs the proliferative response and the mature T cell activation level. T cells from patients with leukemia are functionally impaired, and this is related to decrease TCRζ chain expression [2,3,5,14]. Recently, we has reported the expression pattern of the four CD3 genes in patients with AML and CML [2,4,5,15], and it has been reported that the aberrant TCRζ chain expression found in the T cells of patients with systemic lupus erythematosus (SLE) may be associated with the decreased stability and translation of a TCRζ mRNA with an alternatively spliced 3'-untranslated region [16]. However, the mechanism of TCRζ deficiency in T cells in patients with cancer remains unclear.

The TCRζ gene spans 31 kb in the chromosome 1q23.1 locus and has eight exons that are separated by introns ranging from 700 bp to greater than 8 kb [17,18]. The TCRζ mRNA is a 1,472 kb spliced product of the eight exons with a 492 bp coding region and a long downstream 906 bp 3'-untranslated region (UTR), which is encoded by exon VIII [19]. The stability of the TCRζ mRNA is mainly influenced by the downstream 3’ untranslated region and poly A tail. The 906 bp TCRζ 3'-UTR has several polyadenylation sites. Exon VIII comprises 20 amino acids of the carboxy-terminus and the 3'-UTR of TCRζ chain. Recently, it has been reported that there are several TCRζ chain isoforms with different 3'-UTR nucleotide sequences in mouse T cells [20]. The activation of alternative splicing within the 3'-UTR through two internal (5' and 3') splice sites results in a splice deletion of 562 bases (nucleotides 672–1233), leading to the generation of a 344 bp alternatively spliced (AS) variant [21]. The 344 bp AS TCRζ isoform lacks two critical regulatory adenosine/uridine-rich elements (ARE) and a translation regulatory sequence. The stability and translation of this isoform are significantly lower than that of the 906 bp WT TCRζ isoform; consequently, the relative amount of TCRζ protein generated by the AS isoform is significantly lower than that from the WT isoform [22,23].

T cells from healthy individuals predominantly express the wild type (WT) isoform, whereas an increased level of the AS isoform was reported in T cells from SLE patients [23]. Frequent mutations/polymorphisms and aberrant splicing of the downstream 3'-UTR may affect the stability and/or transport of the TCRζ chain mRNA, leading to its downregulation in SLE T cells [23]. Although differential expression of the TCRζ 3'-UTR isoforms contributes to differential TCRζ protein expression levels, the factor(s) regulating the alternative splicing of the TCRζ 3'-UTR is unknown [19].

Alternative splicing is a powerful gene regulation mechanism that results in the generation of numerous transcripts and proteins from a single gene [24]. Splice site selection is regulated by *cis*-acting elements such as intronic and exonic splicing enhancer and silencer sequences, respectively [25,26]. Alternative splicing factor/splicing factor 2 (ASF/SF2) is a prototypical SR protein that was originally identified in HeLa nuclear extract as a factor required to reconstitute splicing in S100 cellular extract [27,28] and influence alternative splicing site selection in a concentration-dependent manner [28,29]. ASF/SF2 acts early during spliceosome assembly and participates in multiple steps during constitutive splicing and the regulation of alternative splicing by interacting with the pre-mRNAs and/or other splicing factors [28,30,31]. ASF/SF2 regulates the alternative splicing of eukaryotic genes such as caspase-9 and the T cell differentiation marker CD45 [19,32,33]. Recently, the involvement of ASF/SF2 in the post-transcriptional regulation of TCRζ was described in T cells from patients with SLE. ASF/SF2 binds to the 3'-UTR of TCRζ and regulates the shift in alternative splicing from the AS to the WT isoform and regulates TCRζ protein expression [19].

Based on our previous finding that the TCRζ chain gene expression level was significantly decreased in CML, we further investigated the expression pattern of the TCRζ regulating factors TCRζ 3'-UTR and ASF/SF-2, as well as the expression level and correlation of FcεRIγ and ZAP-70, to evaluate the ASF/SF-2 regulating effects of TCRζ 3'-UTR formation.

## Materials and methods

### Samples

Forty newly diagnosed, untreated chronic phase CML patients, including 27 males and 13 females (13–71 years old; median age: 44 years), and 22 patients with CML complete remission ( 16 cases after allo-HSCT and 6 cases after imatinib therapy) were included in this study [6]. The BCR-ABL fusion gene was detected in all samples by RT-PCR. Fourteen healthy individuals including 8 males and 6 females (23–53 years old; median age: 28.5 years) served as controls. Peripheral blood mononuclear cells (PBMCs) were isolated from heparinized venous blood by Ficoll-Paque gradient centrifugation. All procedures were conducted according to the guidelines of the Medical Ethics Committee of the Health Bureau of the Guangdong Province in China.

#### RT-PCR for TCRζ 3′-UTR amplification

Total RNA was isolated from the PBMC samples using the Trizol reagent according to the manufacturer’s protocol (Invitrogen, Carlsbad, CA, USA). First-strand cDNA was synthesized using the random hexamers and reverse transcriptase in the Superscript II Kit (PowerScript Reverse, BD, San Jose, CA, USA) according to the manufacturer’s instructions. The primers for amplification of the TCRζ 3'-UTR and the β2-microglobulin (β2M) gene, which was used as a control, are listed in Table 1. RT-PCR amplification of the TCRζ 3'-UTR was performed as previously described by Nambiar et al. [23].

**Table 1:** List of primers used for PCR analysis

**Primer**	**Sequence(5′--3′)**	**Association number**	**Product size**
TCRζ3^′^-UTR-f	CAGCCAGGGGATTTCCACCACTCAAAG	NM_000734.3	906 bp/344 bp
TCRζ3^′^-UTR-r	CCCTAGTACATTGACGGGTTTTTCCTG		
ASF/SF-2-f	TCTCTGGACTGCCTCCAAGT	NM_006924.4	473 bp/273 bp
ASF/SF-2-r	GGCTTCTGCTACGACTACGG		
TCRζ-f	GCCAGAACCAGCTCTATAAC	NM_009743.3	166 bp
TCRζ-r	TAGGCCTCCGCC ATCTTATC		
FcεRIγ-f	GAGCCTCAGCTCTGCTATATCC	NM_004106.1	172 bp
FcεRIγ-r	TCTCGTAAGTCTCCTG GTGCC		
ZAP-70-f	GTTGACTCATCCTCAGAGACGAAT	NM_001079.3	183 bp
ZAP-70-r	AGGTTATCGCGCTTCAGGAA		
β2M-f	TACACTGAATTCCACCCCCAC	J00105	144 bp
β2M-r	CACTCAATCCAAATGCGGCA		

#### Real-time quantitative reverse transcription–polymerase chain reaction (qRT–PCR)

The expression level of the TCRζ, FcεRIγ, ASF/SF-2, ZAP-70, and β2*-*microglobulin (β2M) genes was determined by SYBR Green I real-time PCR. Briefly, PCR was performed in a 20 μL total volume that contained 1 μL of cDNA, 9 μL of 2.5× SYBR Green I mix (Tiangen, Beijing, China), and 10 μmol/L primer pairs. After an initial denaturation at 95°C for 3 min, 45 cycles consisting of the following procedure was performed using an MJ Research DNA Engine Opticon 2 PCR cycler (BIO-RAD, USA):10 s at 95°C, 30 s at 64°C for β2M and ASF/SF-2, 60°C for TCRζ and ZAP-70, and 40 s at 60°C for FcεRIγ. The relative amount of the genes of interest and the β2M reference gene was measured in two independent assays. The data are presented as the relative expression of the genes of interest relative to the internal control gene as determined by the 2^(−ΔΔCT)^ method [2,34]. Additionally, the specific amplification of the PCR products was analyzed by melting curve analysis and agarose gel electrophoresis. The real-time PCR primers used for all of the gene amplifications were synthesized by Shanghai Biological Engineering Technology Services Co., Ltd. (Table 1).

### Statistical analysis

Univariate analyses were performed using the Mann–Whitney test to compare the means of the differences in the mRNA expression between the CML and healthy control groups. Pearson correlation and linear regression analyses were used to estimate the correlation between age and the mRNA level of the different genes in the different samples using the SPSS 11.5 statistical software. A difference with a *P* < 0.05 was considered statistically significant.

## Results

### TCRζ3′-UTR isoforms in CML

**Figure 1:**
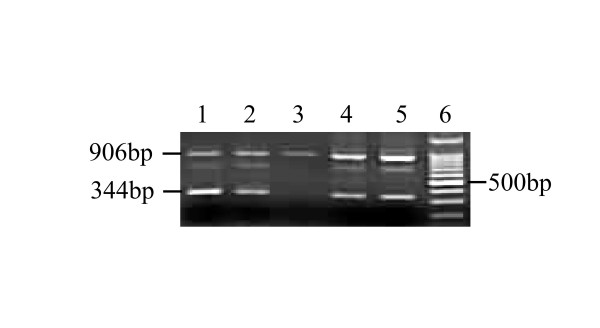
**TCRζ 3'-UTR PCR amplification in healthy individual and CML samples.** Two splice forms, wild type TCRζ 3'-UTR (906 bp) and alternatively spliced TCRζ 3'-UTR (344 bp), could be detected in healthy individuals (lanes 1 and 2), patients with CML-CP (lane 3 and 4) and patients with CML-CR (lane 5). Thirty-five percent of the CML cases contained only the wild type TCRζ 3'-UTR isoform (lane 3). Lane 6 is an 100 bp DNA ladder.

It has been reported that two types of spliceosomes could be found on the TCRζ 3'-UTR [19,23]. In this study, the alternatively-spliced TCRζ 3'-UTR (344 bp) and the wild type TCRζ 3'-UTR (906 bp) could be detected in the same PCR reaction for all healthy individual and CML complete remission (CML-CR) samples (Figure 1). Both PCR products were cloned and sequenced, and the sequence was confirmed by comparison with the sequence found in the NCBI GenBank (data not shown). Both TCRζ 3'-UTR isoforms were also identified in all CML-CR patient samples; however, 35% of the CML cases (14 cases) contained only the wild type TCRζ 3'-UTR (906 bp), and this was significantly different from the healthy individual and CML-CR groups (*p* < 0.001, *p* < 0.001).

### Characteristic expression of ASF/SF-2 in CML

**Figure 2:**
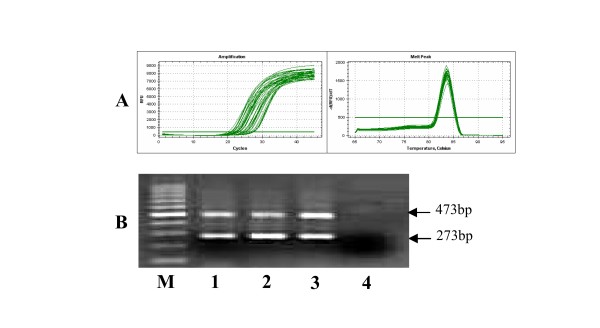
**Melting curve and agarose gel analysis of ASF/SF-2 gene expression. A**: Amplification and Melting curve of the ASF/SF-2 gene by SYBR Green I real-time PCR. **B**: The ASF/SF-2 PCR products were analysis by agarose gel electrophoresis. M: 100 bp DNA ladder, lanes 1–3: samples from the healthy control, CML-CP and CML-CR samples, respectively.

**Figure 3:**
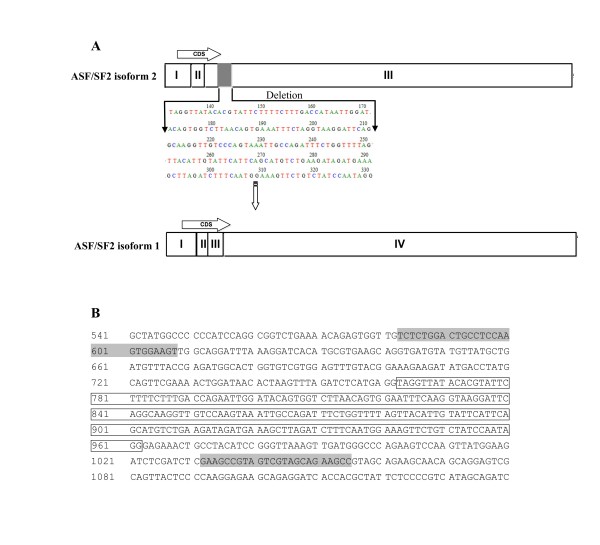
**A: A schematic view of ASF/SF-2 gene bearing the isoform 1 (bottom) and 2 (top). B**: Sequence of the ASF/SF-2 transcript isoform 2 PCR product. The box indicates the 200 bp sequence that was cut in transcript isoform 2, and the gray indicates the PCR primer.

The ASF/SF-2 expression level was quantified by real-time PCR [35]. Although there was only one peak at 83.5°C in the melting curve analysis (Figure 2), two PCR products of 473 and 273 bp were identified by agarose gel electrophoresis (Figure 2). Both of the PCR products were cloned and sequenced, and two specific ASF/SF-2 transcripts were identified by comparison of the sequences in the NCBI GenBank (Figure 3). The 273 bp product was identified as ASF/SF-2 transcript 1, and the 473 bp product was identified as ASF/SF-2 transcript 2 [35]. Thus, the ASF/SF-2 expression level represented the total expression level of all of the ASF/SF-2 transcripts in this study.

### Expression pattern of the ASF/SF-2, TCRζ, ZAP-70 and FcεRIγ genes in CML

The expression level of the TCRζ, FcεRIγ, ASF/SF-2, ZAP-70 genes was determined by real-time PCR using the SYBR Green I technique and quantitatively assessed by comparison with the β2M reference gene using cDNA from PBMCs collected from CML, CML-CR and healthy control samples. Each of the four genes could be detected in every sample. To establish proper real-time quantitative PCR reaction conditions, we used diluted Molt-4 cDNA to make relative standard curves. The results demonstrated that the high amplification efficiency of the four targeted genes was successful and consistent with that of the β2M reference gene. In addition, the specific amplification of the PCR products was confirmed by melting curve and agarose electrophoresis analysis. A single melting curve peak and the expected PCR products were confirmed, the PCR products of all of the genes were randomly selected and sent for sequencing, and the sequencing results were confirmed by BLAST analysis to compare with data in GenBank (data not shown).

**Figure 4:**
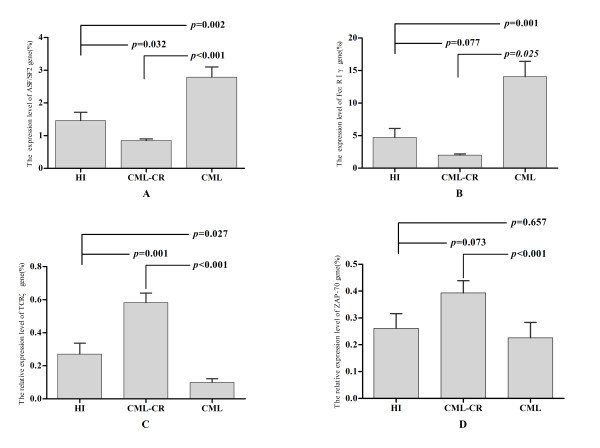
**The expression level of the ASF/SF-2, TCRζ, ZAP-70and FcεRIγ genes in the CML, CML-CR and healthy control groups (HI). A**: ASF/SF-2; **B**: FcεRIγ; **C**: TCRζ; and **D**: ZAP-70.

The expression level of the TCRζ, FcεRIγ, ASF/SF-2 and ZAP-70 genes in PBMCs from CML, CML-CR and healthy controls is shown in Figure 4. The expression of the TCRζ gene in the CML group was significantly lower than that in the healthy control and CML-CR groups (*p* = 0.027,*p* < 0.001), while a significantly higher level of FceRIγ (*p* = 0.001, *p* < 0.001) and ASF/SF-2(*p* = 0.002, *p* < 0.001) gene expression was found in the CML chronic phase (CML-CP) group.

### The expression characteristics of the TCRζ, FcεRIγ, ASF/SF-2 and ZAP-70 genes are related to TCRζ 3'-UTR spliceosome

**Figure 5:**
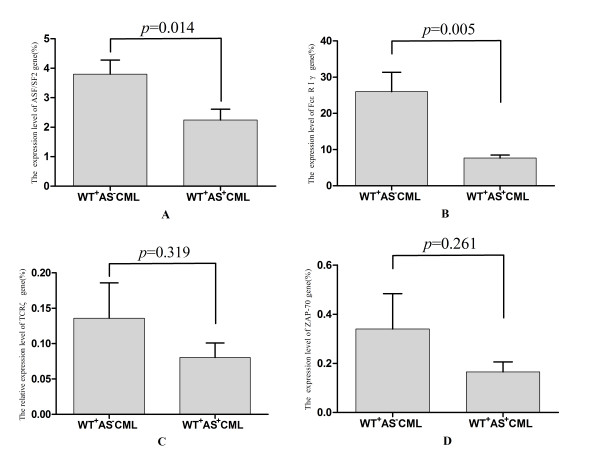
**Differential expression pattern of the ASF/SF-2, TCRζ, ZAP-70 and FcεRIγ genes in the WT^+^AS^- ^and WT^+^AS^+^CML groups. A**: ASF/SF-2; **B**: FcεRIγ; **C**: TCRζ; and **D**: ZAP-70.

To evaluate the effect of the alternatively spliced TCRζ 3'-UTR on the expression and regulation of the TCRζ chain and its related genes according to TCRζ 3'-UTR spliceosome characteristics, patients with CML were divided in two subgroups: patients who only expressed the wild type TCRζ 3'-UTR (the WT^+^AS^-^CML group), and patients who expressed both TCRζ 3'-UTR forms (the WT^+^AS^+^CML group). When the expression level of the TCRζ, FcεRIγ, ASF/SF-2 and ZAP-70 genes was compared between both groups, a significantly higher level of ASF/SF-2 and FcεRIγ gene expression was found in the WT^+^AS^-^CML group (*p* = 0.014, *p* = 0.005), and the level of TCRζ and ZAP-70 gene expression was approximately two-fold higher in the WT^+^AS^-^CML group; however, there was no significant difference in the expression of thses genes when compared with the WT^+^AS^+^CML group (*p* = 0.319, *p* = 0.261) (Figure 5).

### The ASF/SF-2, TCRζ, ZAP-70 and FcεRIγ gene expression is correlated in CML

**Figure 6:**
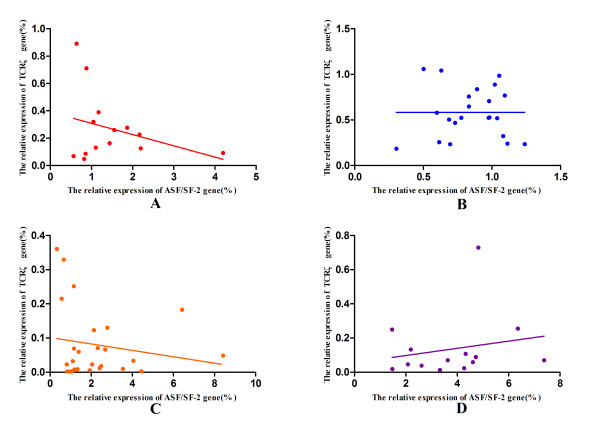
**Correlation analysis of the TCRζ and ASF/SF-2 genes. A**: Healthy controls; **B**: CML-CR group; **C**: WT^+^AS^+^ CML group; and **D**: WT^+^AS^-^ CML group.

ASF/SF-2 regulates TCRζ expression by binding to the TCRζ 3’-UTR and down-regulating the alternatively-spliced TCRζ 3’-UTR isoform. To further investigate the mechanism for lower TCRζ expression in CML, we analyzed correlations between the relative ASF/SF-2, TCRζ, ZAP-70 and FcεRIγ gene expression levels. A negative-correlation was observed between the expression level of TCRζ and ASF/SF-2 expression, but the difference was not statistically significant (r = −0.314, *p* = 0.275). In contrast, the negative correlation between these genes was lost in the CML-CR and CML groups (r = 0.001, *p* = 0.997; r = 0.076, *p* = 0.642, respectively). When we further analyzed the correlation of the TCRζ and ASF/SF-2 gene expression in the WT^+^AS^-^ and WT^+^AS^+^ CML groups, we found that a similar negative-correlation could be observed in the WT^+^AS^+^ CML group (r = −0.17, *p* = 0.407), and no correlation was observed in the WT^+^AS^-^ CML group (r =0.198, *p* = 0.497) (Figure 6).

**Figure 7:**
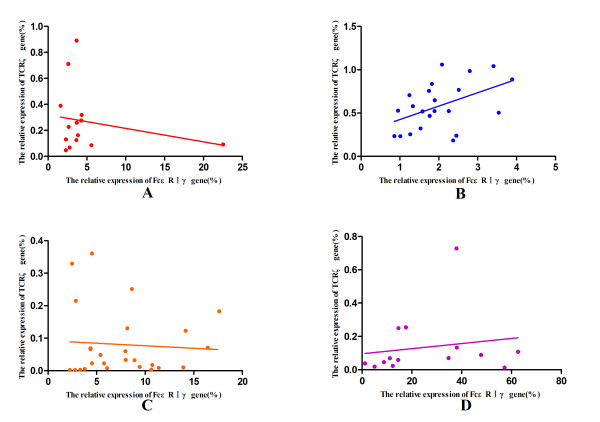
**Correlation analysis of the TCRζ and FcεRIγ genes. A**: Healthy controls; **B**: CML-CR group; **C**: WT^+^AS^+^ CML group; and **D**: WT^+^AS^-^ CML group.

Similar to a previous finding [36], a negative correlation between the level of TCRζ and FcεRIγ gene expression was found in the healthy group, though the difference was not statistically significant (r = −0.218, *p* = 0.454), and this negative correlation was lost in the CML group (r = 0.076, *p* = 0.642); however, the negative-correlation remained in the WT^+^AS^+^ CML group (r = −0.066, *p* = 0.748), and no correlation was observed between these genes in the WT^+^AS^-^ CML group (r =0.165, *p* = 0.573). In contrast, a significant positive correlation of the expression of both genes was found in the CML-CR group (r = 0.473, *p* = 0.026) (Figure 7).

**Figure 8:**
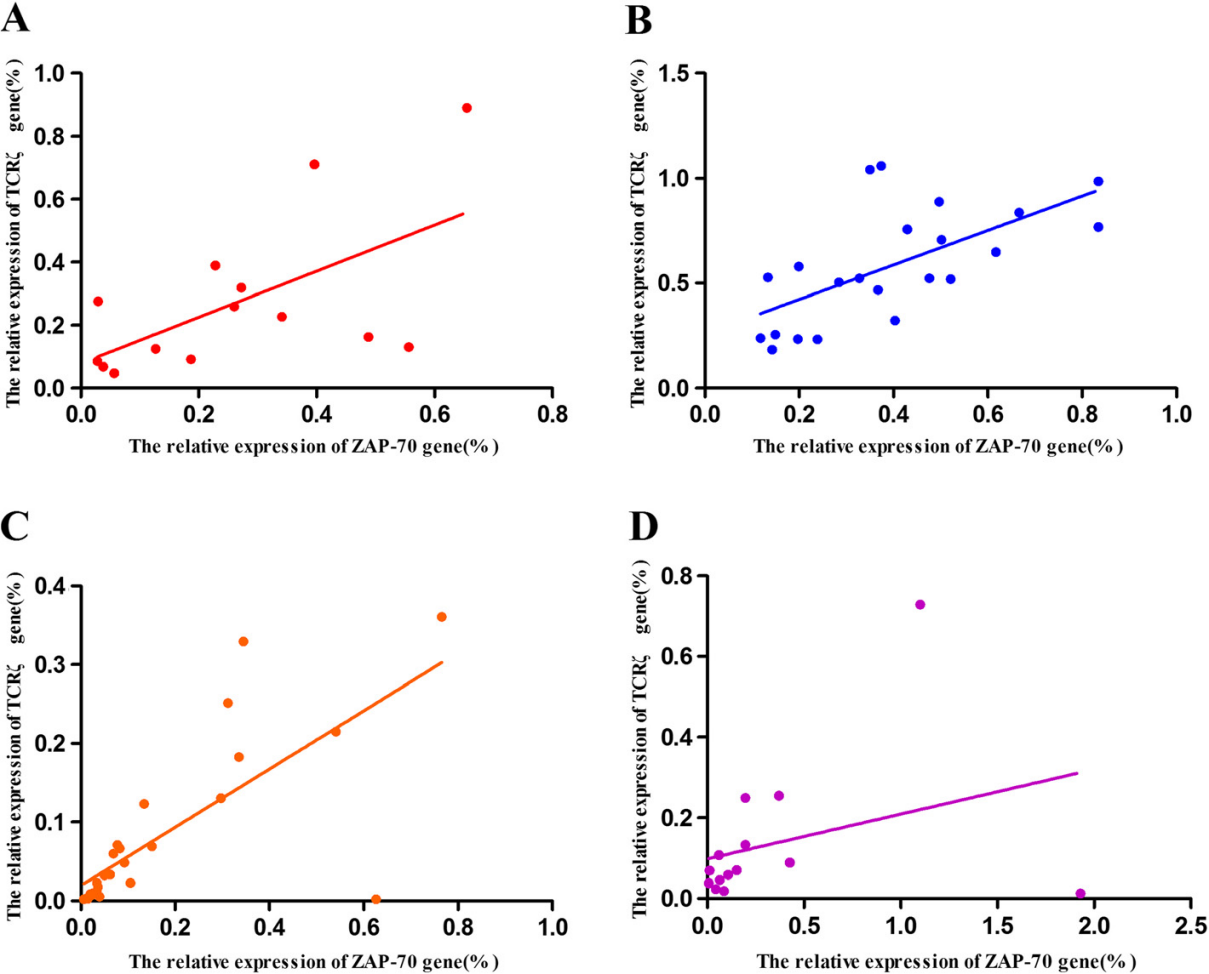
**Correlation analysis of the TCRζ and ZAP-70 genes. A**: Healthy controls; **B**: CML-CR group; **C**: WT^+^AS^+^ CML group; and **D**: WT^+^AS^-^ CML group.

The expression correlation of the TCRζ and ZAP-70 genes was analyzed, and a significant positive correlation was found in the healthy control, CML-CR and CML groups (r = 0.600, *p* = 0.023; r = 0.637, *p* = 0.001; and r = 0.460, *p* = 0.003, respectively). The same result was also found in the WT^+^AS^+^ CML group (r =0.737, *p* < 0.001), while no significant correlation was found in the WT^+^AS^-^ CML group (r =0.320, *p* = 0.265) (Figure 8).

A significant positive correlation of the ASF/SF-2 and FcεRIγ expression level was found in the healthy control and CML groups (r = 0.822 *p* < 0.001 and r = 0.334, *p* = 0.035, respectively).

## Discussion

### Defects inTCR/CD3 signaling in CML

The TCR/CD3 complex plays a central role in T cell activation, and the alteration of any subunits in the complex may change the T cell activation level [7-10]. An abnormal TCR repertoire, lower thymic output function and lower CD3 gene expression have been described in CML [2-6]. Alternative CD3 gene expression levels may directly represent a characteristic of lower T cell activation [2-4]. In this study, the TCRζ expression level was detected in 40 patients with chronic phase CML and compared with CML in complete remission and healthy individuals. Similar to our previous study, we found that the TCRζ gene expression level in the CML group was significantly lower than that in healthy controls [2], and we further demonstrated that the TCRζ gene expression level may be increased in patients with CML who achieved complete remission. It is possible that the high number of CML cells in the blood may have influenced the results; however, our previous studies have demonstrated decreased expression of the gene and protein encoding the TCRζ chain in purified CD3 + T cells in chronic phase CML by quantitative real-time PCR and FCM, respectively [37]. Thus, we used PMBC samples as a simple method to analyze the expression characteristics of the TCRζ chain and its related genes.

Unlike the TCRζ chain, which mediates signaling through ZAP-70, FcεRIγ mediates signaling by associating with the phosphorylated protein kinase *Syk,* which is 100-fold more potent than ZAP-70 and is preferentially recruited to the FcεRIγ receptor [12,38-40]. Thus, FcεRIγ may substitute for deficiencies in the TCRζ chain. In this study, we analyzed the FcεRIγ gene expression level and its correlation with TCRζ gene expression in patients with CML. As expected, the FcεRIγ expression level was significantly increased in patients with CML, while its expression level was lower in patients who achieved complete remission. These data suggest that, in the context of CD3ζ down-regulation, FcεRIγ expression in CML is up-regulated to contribute to TCR signaling transduction in a manner similar to that of the conserved functional ITAM motif, which is a different immune status than that found in CLL, where the FcεRIγ expression level was not up-regulated and was not correlated with the TCRζ expression level [36]. However, FcεRIγ expression level did not demonstrate negative correlation with TCRζ expression level in patients with CML, suggesting that apparent defects in T cell-mediated immunity are involved in alternative immune regulation in CML. When we further analyzed the correlation in the WT^+^AS^+^ and WT^+^AS^-^ CML groups, we found that the negative-correlation remained in WT^+^AS^+^ CML group, and by combining the gene expression characteristics of the WT^+^AS^-^ CML group, it is thought that the defects in immune regulation is apparent in the WT^+^AS^-^ CML group due to the differential distribution of TCRζ-3^′^UTR spliceosomes. An interesting and significant positive correlation between the expression of TCRζ and FcεRIγ genes was found in the CML-CR group, and whether this is the result of an abnormal immune regulation status rather than immunodeficiency in patients with CML-CR remains an open question.

It is well known, ZAP-70 is a downstream factor that transduces TCR signals [41]. In this study, the finding of a significant positive correlation between the expression level of the TCRζ and ZAP-70 genes in the healthy control, CML-CR and CML groups further supports the correlation of the TCRζ and ZAP-70 genes in T cell activation.

### The molecular mechanism of decreased TCRζ expression in CML

A lower transduction of the TCR signal may be a common feature in hematological malignancies [36]. The absence of the TCRζ chain not only influences the TCR expression on the cell membrane and the number of single-positive (CD4+ or CD8+) circulating T cells, it also impairs the proliferative response and mature T cell activation level [42,43]. However, the mechanism of TCRζ deficiency in T cells in patients with cancer is unclear. To gain insight into the molecular mechanism of TCRζ deficiency in CML, we analyzed the distribution of the TCRζ 3'-UTR isoforms, which contribute to the regulation of TCRζ expression [19], and the ASF/SF-2 gene expression level, which regulates the alternative splicing of eukaryotic genes.

In general, the TCRζ mRNA stability is mainly influenced by its downstream 3'-UTR. While the 906 bp WT 3'-UTR plays an important role in TCRζ transcript stability, the 344 bp alternatively spliced 3'-UTRsignificantly influences the generation of the TCRζ [22,23]. Interestingly, while we found that 35% of the CML samples in this study contained only the wild type TCRζ 3'-UTR isoform, the wild type and alternatively spliced TCRζ3'-UTRs isoform could be detected in all healthy individual and CML-CR samples. These results may account for the feedback regulation of the immune system in certain CML cases. Moreover, samples that contained only the wild type TCRζ 3'-UTR isoform demonstrated a high expression level for the ASF/SF-2, FcεRIγ, TCRζ and ZAP-70 genes, and based on this finding, we tried to characterized the different CML subgroups using the different expression patterns of the TCR signaling components. A definitive characteristic was found when comparing the gene expression pattern of the ASF/SF-2, FcεRIγ, TCRζ and ZAP-70 genes in the WT^+^AS^-^and WT^+^AS^+^ CML groups.

The involvement of ASF/SF2 in the post-transcriptional regulation of TCRζ was described in T cells from patients with SLE. ASF/SF2 binds to the 3'-UTR of the TCRζ mRNA and regulates a shift in alternative splicing from the AS to the WT isoform, which modulates TCRζ protein expression [19]. Therefore, a higher ASF/SF2 expression level is correlated with a higher WT TCRζ3'-UTR expression level, which results in a higher TCRζ protein expression level. In this study, a significantly higher ASF/SF2 expression level was found in patients with CML who had a lower TCRζ level, prompting the question of whether there is feedback regulation in patients with CML similar to that for the enhanced FcεRIγ expression level. However, this feedback regulation was unable to recover the TCRζ expression level. Interestingly, significantly higher ASF/SF2 expression was found in the WT^+^AS^-^group as compared with the WT^+^AS^+^CML group, a result that is in agreement with the biological findings. No study has reported similar results; thus, further investigation of more samples with outcome follow up for patients with CML is required in future studies.

In conclusion, to our knowledge, this is the first study attempting to provide a global gene expression profile of the TCRζ related genes: FcεRIγ, ASF/SF-2 and ZAP-70, and the distribution characteristics of the TCRζ 3'-UTR isoforms in de novo CML and CML-CR patients with TCRζ defective. The preliminary data may indicate that defective TCRζ expression may be characterized in the WT^+^AS^-^ and WT^+^AS^+^ CML subgroups with different gene expression patterns. The TCRζ 3'-UTR alternative splicing characteristics may be a novel immunological marker for the evaluation of the CML immune status. Moreover, ASF/SF-2 may also be a target for regulation by the immune system to overcome immunodeficiency in CML.

## Competing interests

Authors have no potential competing interest.

## Authors’ contributions

YQL contributed to concept development and study design. XFZ, XJY, QS, XLW, SHC, BL and LJY performed the laboratory studies. YPZ, SXG, JYW and XD were responsible for collection of clinical data. YQL, XFZ and XJY coordinated the study and helped drafting the manuscript. All authors read and approved the final manuscript.
